# Fitness consequences of trait‐mediated plant–pollinator interactions

**DOI:** 10.1002/ajb2.70167

**Published:** 2026-02-21

**Authors:** Christine S. Sheppard, Frank M. Schurr, Ingo Grass

**Affiliations:** ^1^ Institute of Landscape and Plant Ecology University of Hohenheim Stuttgart 70593 Germany; ^2^ Center for Biodiversity and Integrative Taxonomy (KomBioTa) University of Hohenheim Stuttgart 70593 Germany; ^3^ Institute of Botany, Department of Ecosystem Management, Climate and Biodiversity BOKU University Gregor‐Mendel‐Straße 33 Vienna 1180 Austria; ^4^ Ecology of Tropical Agricultural Systems University of Hohenheim Stuttgart 70593 Germany

**Keywords:** competition, facilitation, fitness landscape, *Impatiens glandulifera*, plant invasion, plant–pollinator interaction, trait matching

Most angiosperm species depend at least partly on animal pollinators; when pollinators are excluded, a third produce no seeds, and half produce at least 80% fewer seeds (Rodger et al., 2021). Simultaneously, many animal pollinators strongly depend on floral resources for survival and reproduction. Quantifying fitness consequences of plant–pollinator interactions holds great promise for understanding ecological and evolutionary dynamics and for assessments of global change impacts. To this end, we suggest a novel approach using fitness landscapes of plant–pollinator interactions, which we illustrate by using the example of human‐mediated invasion by alien species.

## PLANT–POLLINATOR INTERACTIONS INVOLVING INVASIVE ALIEN PLANTS

Invasive alien species are a major threat to biodiversity. When alien plant species become dominant, they can fundamentally alter plant–pollinator interactions (Parra‐Tabla and Arceo‐Gómez, 2021) and thus provide a natural experiment to unravel the mechanisms governing fitness consequences of species interactions. Although many alien plants rely on local pollinators to invade new areas, pollinator limitation is not usually thought to limit invasions. In some cases, pollinators may be introduced with the plant invaders (e.g., fig wasp pollinators with alien *Ficus* species), but more often the alien species will co‐opt generalist pollinators of the same functional types as their pollinators in the native range (Stout and Tiedeken, 2017). Furthermore, floral traits of invasive plant populations may rapidly evolve in response to local pollinator pools (Schiestl, 2024).

Pollinators in turn may benefit from a new species that provides abundant floral resources (e.g., Chittka and Schürkens, 2001). On the other hand, the tendency of many invasive plants to form dense monocultures that provide only one type of floral resource can threaten pollinator diversity. The impacts of invasive plants on pollinators may also affect their indirect interactions with native plants (Morales and Traveset, 2009; Parra‐Tabla et al., 2025). Pollinator‐mediated facilitation of native plants occurs if the invader acts as a “magnet species”, attracting many pollinators and causing spillover effects on native plants. By contrast, pollinator‐mediated competition occurs when invaders weaken mutualistic interactions between native plants and pollinators. Importantly, fitness of native plants at invaded sites may not just be lower because natives receive fewer pollinator visits, but also because the quality of visits declines with a lower ratio of conspecific to heterospecific pollen transfer (e.g., Parra‐Tabla et al., 2025), an aspect that most studies focusing only on pollinator visitation rates do not consider.

## FITNESS LANDSCAPES OF PLANT–POLLINATOR INTERACTIONS

Given the contradictory evidence on how invasive alien plants impact the fitness of pollinators and co‐occurring native plant species, a trait‐based approach is a promising avenue because the traits governing plant–pollinator interactions are generally well understood. Functional traits are those characteristics of organisms that impact fitness indirectly via their effect on survival, growth, or reproduction (Violle et al., 2007). Trait matching occurs when trait values of interacting partners fit well, such as the matching of flower morphology (e.g., flower depth) and pollinator mouthparts (e.g., proboscis length; Stang et al., 2006) that allows the transfer of pollen and uptake of nectar during the flower visit. Such trait matching may help to explain interaction probability and strength (e.g., Naghiloo et al., 2021) and the resulting fitness consequences for the interacting partners. We here focus on mutualistic plant–pollinator interactions, although we note that plant–pollinator interactions can range from mutualistic to antagonistic (e.g., nectar robbers, Van der Kooi et al., 2021). Regarding pollinator‐mediated interactions between plants, the similarity of floral traits of alien and native plant species can determine how the fitness of plant species and pollinators changes with the alien invasion (Parra‐Tabla et al., 2025). Both similarity in floral traits related to visitation rates, such as flower depth and size, and traits related to the likelihood of heterospecific pollen deposition, such as pollen size and style length, may affect native plant fitness.

**Figure 1:**
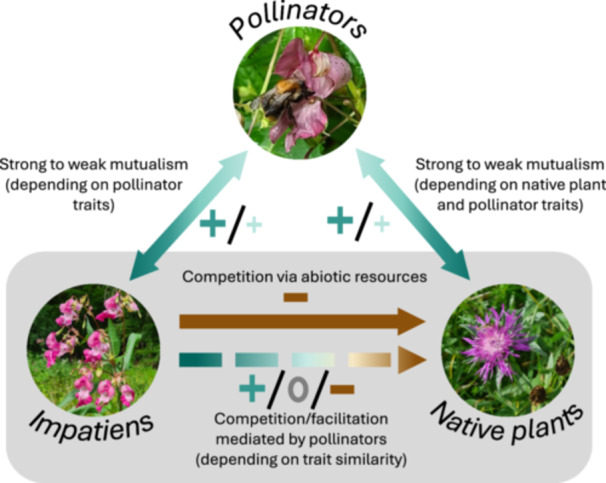
Possible interactions between a dominant invasive alien plant such as *Impatiens glandulifera*, native plants, and pollinators (photographs © C. S. Sheppard). Note that in this essay, we focus on mutualistic plant–pollinator interactions and the indirect consequences for native plant–invader interactions only.

As an example, we consider the invasion by *Impatiens glandulifera* (Himalayan balsam), a well‐known invasive species in Europe, North America, and New Zealand. Its flowers produce a lot of sugar‐rich nectar and attract many pollinators, particularly bumblebees, which can reduce pollinator visits to and seed set of co‐occurring native plants (Chittka and Schürkens, 2001). The interactions of *I. glandulifera* with native plants and pollinators are expected to range from negative to positive depending on the trait similarity and matching of native plants and pollinators (Figure 1).

To understand and predict the fitness consequences of the interaction triangle depicted in Figure 1, we propose using the concept of fitness landscapes of biotic interactions (FLINTs), which are “maps that relate the per‐capita effect of an interaction partner on the fitness of a focal organism … to traits of both the focal organism and the interaction partner” (Schurr et al., 2025, lines 81–83 [preprint]). For constructing such maps for the given study system, the most important interaction partners are determined (e.g., the native plant species and relevant pollinator species in the habitat invaded by *I. glandulifera*), for which the most important trait(s) mediating the interaction are then measured. An experimental setting with standardized conditions is ideal to determine the per‐capita fitness consequence of each pairwise interaction. The fitness of each interaction partner both in the presence and the absence of the interaction is measured and the differences with vs. without the interactions are mapped (Schurr et al., 2025 [preprint]). The resulting fitness landscape of plant–pollinator interactions then illustrates the fitness consequences for all possible combinations of interaction partners, mediated by the varying degrees of trait matching.

**Figure 2:**
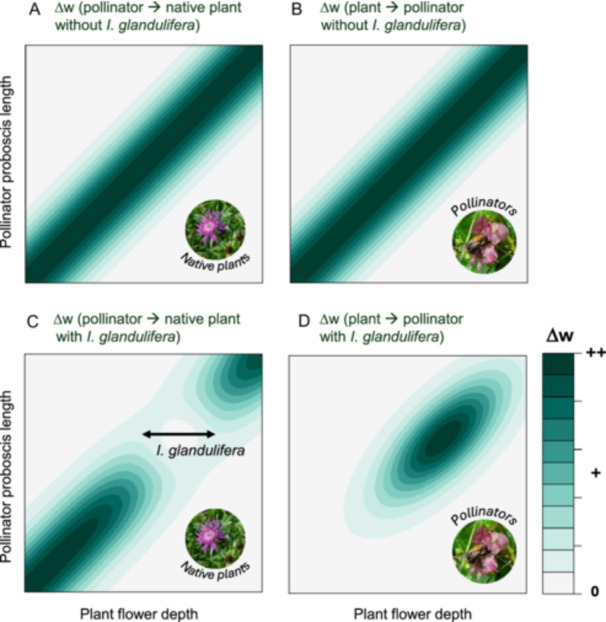
Fitness landscapes of mutualistic plant–pollinator interactions depicting hypothesized consequences for the fitness of native plants and pollinators. These fitness landscapes show how fitness consequences (Δw; that is the difference in fitness with vs. without the plant–pollinator interaction) depend on plant and pollinator traits (e.g., flower depth and proboscis length). Native plant fitness (A, C) and pollinator fitness (B, D) are shown in the absence (A, B; trait matching) and in the presence (C, D) of a dominant invasive alien plant such as *Impatiens glandulifera*. The inserted photographs (© C. S. Sheppard) illustrate whether the fitness landscape refers to the fitness of native plant or pollinator species, but note that they represent just one example of a relevant native plant and pollinator species in this system.

In the case of simple trait matching, maximum fitness is reached when traits such as pollinator proboscis length and plant flower depth match perfectly (Figure 2A, B). In more complex real‐world scenarios (such as disruptions of plant–pollinator interactions due to the arrival of alien species, depicted in Figure 1), simple trait matching between native plants and pollinators is likely not sufficient to predict the fitness consequences of such interactions. In this scenario, we hypothesize, that if invasive alien species and natives compete for the same pollinators, native species with similar floral traits are most affected (Figure 2C). Accordingly, negative effects of aliens on co‐occurring natives are strongest when alien and native plants have similar flower symmetry or color (Morales and Traveset, 2009, but see Charlebois and Sargent, 2017). In such a case, a depression in the fitness landscape appears for native plants that are too similar to *I. glandulifera* (Figure 2C). Furthermore, native pollinators that have traits matching the invasive plant benefit; the others disappear (Figure 2D).

## EXPERIMENTAL APPROACHES TO STUDY FITNESS LANDSCAPES OF PLANT–POLLINATOR INTERACTIONS

Our ability to predict pairwise plant–pollinator interactions is still limited (Peralta et al., 2024). Most studies of plant–pollinator interactions with invasive plants are phenomenological rather than mechanistic, such as observational studies of plant–pollinator communities and their interaction networks in nature. Crucially, to understand an invader's role and its impacts in plant–pollinator networks, studies need to go beyond assessing visitation rates, networks, and community descriptions, instead considering fitness consequences of interactions and the resulting population dynamics (Parra‐Tabla and Arceo‐Gómez, 2021). When focusing on fitness consequences, we need to consider the effects of varying pollinator visitation and of heterospecific pollen deposition.

Naturally, quantifying fitness landscapes of biotic interactions comes with many challenges, such as how to identify relevant traits and how to measure fitness (Schurr et al., 2025 [preprint]). Experimental assembly of plant–pollinator communities is a powerful approach to study fitness landscapes of plant–pollinator interactions, for instance using mesocosms, a highly controlled yet semi‐natural setting in which the experimental factors of interest can be manipulated (Brendel et al., 2023). At the same time, abiotic conditions and initial density of interacting partners can be standardized. Larger mesocosms that allow the enclosure of specific pollinators and plants in the field (e.g., Klaus et al., 2021) can be employed, growing an invasive alien plant with many different combinations of native plant and pollinator species of varying traits. Plant fitness can then be quantified, for instance, for annual plants as total seed set (Brendel et al., 2023) and subsequent seed germination, and for perennial plants by quantifying individual demographic rates fed into integral projection models (Bialic‐Murphy et al., 2020). We note, however, that these approaches only quantify female fitness. The fitness of each studied pollinator species can be quantified on the basis of reproductive rates, such as by examining the number of offspring of solitary bees, from brood cell establishment to the number and sex of adults in the subsequent generation (Klaus et al., 2021), or for social bees by assessing colony growth and the number of overwintering queens at the end of the colony cycle (Westphal et al., 2009). Another challenge is that the density of interacting partners likely has a strong effect. To fully understand the fitness consequences of real‐world interactions, one would then also need to quantify the numerical response of interaction effects, that is, how the interaction effects scale with the densities of the interaction partners (Schurr et al., 2025 [preprint]). Although logistically challenging, such approaches would allow mapping of fitness landscapes of plant–pollinator interactions and thus experimentally test the hypothesized relationships of Figure 2C, D.

## CONCLUSIONS

We propose using the concept of fitness landscapes of plant–pollinator interactions to advance our fundamental understanding of how species interactions shape ecological dynamics. The arrival of invasive alien species, with their direct and indirect effects on native plants and pollinators, which are often mediated by functional traits, offers a natural experiment for exploring these dynamics. The concept of fitness landscapes of plant–pollinator interactions also applies to dominant plant species that are not of alien origin (e.g., range shifts due to climate change). More broadly, the fitness landscapes concept can be extended to other types of biotic interactions (e.g., plant–herbivore; Schurr et al., 2025 [preprint]). Such conceptual and methodological advances are crucial for improving predictions of plant–pollinator interactions (Parra‐Tabla and Arceo‐Gómez, 2021; Peralta et al., 2024) and for assessing how global change affects population dynamics and ecosystem services.

## AUTHOR CONTRIBUTIONS

C.S.S., F.M.S., and I.G. developed the concepts; C.S.S. wrote the first draft of the manuscript with input and contributions from all authors. All authors acquired funding.

## Data Availability

No data were created or analyzed in this study.
